# Supplementary material for: Quality of life in hypertensive patients using the WHOQOL-BREF instrument in the post-pandemic Bangladesh: A cross-sectional study
Source: PLoS One. 2026 Jan 16;21(1):e0340897. doi: 10.1371/journal.pone.0340897 (PMC12810801; doi:10.1371/journal.pone.0340897)
Supplement: S1 File — (PDF) [file pone.0340897.s002.pdf]

Memo No: NUB/ DPH/EC/ 2024/ 34

Date: 27 February 2024

To
Dr. Nasrin Akter
Lecturer
Department of Public Health
Northern University Bangladesh

Subject: Ethical Approval

With reference to your application dated 15 February 2024 on the above subject, this is to inform you that your research proposal titled "**Quality of Life in hypertensive patients using the WHOQOL-BREF instrument in post-pandemic Bangladesh: A cross-sectional study followed analytical approach**" has been reviewed and approved by the Ethical Review Committee of Department of Public Health, Northern University Bangladesh (NUB) on condition that the guidelines overleaf are strictly followed during execution of the study.

Dr. Monowar Ahmad Tarafdar
Chairman, Ethical Review Committee
Department of Public Health, NUB

The ethical guideline to be followed by the principal and co-investigators

- The right and welfare of individual volunteer are adequately protected.
- The methods to secure informed consent are fully appropriate (in the case of minors, consent should be obtained from parents and guardians) and adequate measures are taken to safeguard confidentiality of information.
- The investigator(s) assume the responsibility of modifying to the ethical review committee of any change in methodology of the protocol whether or not any risk by the individual volunteers is involved.
- To immediately report to ethical review committee if any evidence of unexpected or adverse reaction is noted in the subject under study.
- The proposal is subjected to PI’s reading and accepting the ethical review Principle’s and Guidelines are currently on operation.

111/2 Kawlar Jame Mosjid Road, Dakshin Khan, Dhaka-1230
Tel : +(880-2) 22227593-95, 22227718-20, Fax : +(880-2)9820720
FB/NUBDhaka, E-mail: info@nub.ac.bd, Website: www.nub.ac.bd
