# Supplementary material for: Quality of life in hypertensive patients using the WHOQOL-BREF instrument in the post-pandemic Bangladesh: A cross-sectional study
Source: PLoS One. 2026 Jan 16;21(1):e0340897. doi: 10.1371/journal.pone.0340897 (PMC12810801; doi:10.1371/journal.pone.0340897)
Supplement: Attachment — Submitted filename: Plos One_Response to Reviewer Comments_01.07.25.docx [file pone.0340897.s006.docx]

**Review Comments to the Author**

Please use the space provided to explain your answers to the questions above. You may also include additional comments for the author, including concerns about dual publication, research ethics, or publication ethics. (Please upload your review as an attachment if it exceeds 20,000 characters)

**Reviewer #1:** The manuscript focuses on a health issue that is highly relevant for research and practical purposes.

1. Although the manuscript is clear and for the most part coherent, the novelty aspect is quite weak. Thus, I find it hard to recommend it for publication in its current form. It seems that the gap is related to the lack of research to "provide culturally relevant insights to guide effective healthcare interventions and improve the well-being of individuals living with hypertension." The authors did not make it clear how the existing literature is lacking in this matter. Additionally, how does measuring QOL would fill this gap? What other alternatives are there to solve this problem? Is compiling QOL data the most effective way to solve the problems? I highly doubt the assertion that "Without this data, it’s hard to develop effective and culturally appropriate strategies to support and improve the well-being of hypertensive patients". There are already a strong body of knowledge to be used. If it is indeed culturally inappropriate for Bangladeshi context, then the authors have to explain so.

A stronger and clearer argument need to be written to highlight the novelty of this study.

Thank you for your feedback. To address this, we have revised the last two paragraphs of the Background section to more clearly articulate the novelty of our study. We clarified that while there is existing global knowledge on QOL in chronic conditions, studies specifically using WHOQOL-BREF in hypertensive populations in post-pandemic Bangladesh remain limited. We also justified why QOL data is critical for designing culturally relevant strategies—something not possible with generalized data from other diseases or global contexts (Line 81-85). As noted in the discussion section, during the pandemic, the European Quality of Life-5 Dimensions (EQ-5D) tool was used to assess quality of life among hypertensive patients in Indonesia. To address your query about alternatives, we added a comparison of other methods such as EQ-5D and HRQoL tools and justified why WHOQOL-BREF was selected. To further contextualize our choice of instrument, the following paragraph is added (Line no 76-84)-

“While tools such as the EQ-5D are widely used for assessing QOL, they primarily focus on health utility values and are more suitable for economic evaluations. In contrast, WHOQOL-BREF offers a broader, multidimensional assessment—including physical, psychological, social, and environmental domains, making it more appropriate for understanding the lived experiences of hypertensive patients in a culturally nuanced context. Additionally, the WHOQOL-BREF has been cross-culturally validated and shown to be adaptable for use in LMICs like Bangladesh, supporting its relevance for this study”.

2. From a research literature perspective, it is not clear why hypertensive patients were chosen as the focus of the study. How are the patients different enough compared to other patients? Why can't the authors simply use existing studies on QOL of patients with various illnesses to "develop effective and culturally appropriate strategies".

Thank you for your insightful observation. We have now explicitly clarified the rationale for selecting hypertensive patients as the focus of our study. Although QOL research exists for patients with various chronic illnesses, hypertension presents distinct clinical and psychosocial challenges. As a chronic asymptomatic condition with serious long-term consequences, hypertension often lacks adequate patient engagement, leading to reduced treatment adherence and quality of life. Furthermore, the psychological and social impacts of hypertension, particularly in post-pandemic Bangladesh, remain under-researched, especially using multidimensional tools like WHOQOL-BREF. Our revised manuscript now explains why general QOL studies cannot be directly applied to hypertensive populations without cultural and contextual adaptation. This justification is now reflected clearly in the background section. The following paragraph is added in the background section (Line no 86-96)-

“While QOL research among chronic illnesses exists, hypertension warrants distinct attention due to its asymptomatic nature and long-term behavioral management demands. Unlike conditions that present with immediate symptoms (e.g., diabetes or stroke), hypertension’s “silent” progression can result in patients underestimating its seriousness, thereby diminishing adherence to treatment and engagement with health services. Moreover, the post-pandemic psychosocial stressors—such as social isolation, fear of comorbid risk, and disrupted routines—may uniquely impact individuals with hypertension. Thus, generalized QOL findings from other illness groups may not capture the nuanced experiences of hypertensive patients in Bangladesh. This study specifically addresses this gap by exploring their QOL through a multidimensional and culturally adaptable tool”.

3. Conclusion section: "As predictor, poor QOL in all domains were found significant among the older, diabetic patients who had history of COVID-19, and poor monthly family income." I have 2 concerns about this conclusion:

a. If the authors want to address cultural nuances, how does these significant predictors relate to cultural uniqueness of Bangladesh? In other words, what culture specific findings are there?

b. How will these findings help in designing effective strategies? Increase the patients' income? Make them younger? Cure their diabetes? I can't see how the identification of factors influencing QOL can help in designing such strategies.

Thank you for raising these thoughtful concerns. We agree that the cultural relevance of our findings and their practical implications should be more explicitly stated. The predictors—older age, diabetes, COVID-19 history, and lower income—are not just biological or economic markers; they intersect with social norms, access to care, and health-seeking behaviors in Bangladesh. For example, older adults in extended families may prioritize others’ health over their own, and stigma around chronic disease or mental health can deter engagement with care. Limited financial resources often restrict dietary options and continuity of treatment. Thus, these predictors reflect cultural and structural vulnerabilities that influence health outcomes, rather than fixed individual attributes.

Rather than “making patients younger” or “curing diabetes,” our intent is to guide culturally tailored strategies—like age-appropriate psychosocial support, community-based diabetes education, or income-sensitive intervention packages (e.g., subsidized nutrition counseling or mobile health services). These findings help stakeholders identify high-risk subgroups for targeted resource allocation, and they emphasize the need to go beyond pharmacologic care by addressing social determinants within culturally meaningful frameworks.

The following paragraph is added (Line 455-458):

Poor QOL across all domains was significantly associated with older age, diabetes, prior COVID-19 infection, and lower monthly family income. These factors reflect not only clinical vulnerability but also cultural and socioeconomic conditions in Bangladesh, such as limited access to care, dietary constraints, and caregiving roles within multigenerational families.

Again, the following paragraph is added (Line 460-463):

These findings appeal the need for culturally grounded strategies, including family-based health education, peer support for older adults, and subsidized services for low-income patients, to enhance patient-centered care beyond clinical treatment.

4. The method needs more details (e.g. what kind of random selection method was used? how long had the patients have hypertension? What are the levels of hypertension experienced by the patients? What language was used for the WHOQOL?)

We appreciate your valuable feedback. In response, we have revised the Methods section to clarify the random sampling technique, participants’ duration of hypertension, classification of hypertension levels, and the language used in administering the WHOQOL-BREF instrument.

Specific Revisions Made:

1. For addressing random Selection Method concern the following below is added in section 2.2 (Line 152-155)

“Initially, 600 hypertensive inpatients were identified from the admission registry of the Cardiac & Vascular Surgery and Cardiology (Interventional) departments. From this pool, every second patient was selected using systematic random sampling, resulting in a final sample of 300 participants.”

2. For addressing duration of Hypertension concern the following below is added in section 2.2 (Line 133-135)

“Participants had been diagnosed with hypertension for a minimum duration of six months prior to the survey, based on hospital records.”

3. For addressing levels of Hypertension concern the following below is added in section 2.3 (Line 184-186)

“Hypertension levels were categorized using JNC 8 criteria into Stage 1 (systolic 140–159 mmHg or diastolic 90–99 mmHg) and Stage 2 (systolic ≥160 mmHg or diastolic ≥100 mmHg) based on recent clinical measurements retrieved from patient charts.”

4. For language of WHOQOL-BREF – The following below is added in section 2.3 (Line 179-181)
   “The WHOQOL-BREF was administered in Bengali, the native language of participants, ensuring cultural and linguistic appropriateness.”
5. What previous validity evidence are there regarding the use of WHOQOL-BREF among Bangladeshi? Internal consistency index (Cronbach alpha) is not a strong evidence for the validity of the scores. Analyses like CFA or Rasch Rating Scale Model are more robust to convince readers of the validity of the scores.

We sincerely thank you for the valuable comment. While our study reported satisfactory internal consistency using Cronbach’s alpha across the WHOQOL-BREF domains, we acknowledge that this metric is not strong evidence for the validity of the scores. In response, we have revised the following paragraph (line no 412- 420)-

“In the Bangladeshi context, prior studies have supported the construct validity of the WHOQOL-BREF using more robust statistical methods. For example, Amin et al. (2022) validated the tool among type 2 diabetes patients using confirmatory factor analysis (CFA), while Islam (2025) applied both CFA and item-level analyses to confirm the instrument’s multidimensional structure among older adults. These findings reinforce the psychometric soundness of the WHOQOL-BREF for use in diverse Bangladeshi populations. However, we agree that future research employing Rasch analysis or more comprehensive CFA models in hypertensive populations would further strengthen the evidence base for its use in this context”.

References:

Amin MF, Bhowmik B, Rouf R, Khan MI, Tasnim SA, Afsana F, Sharmin R, Hossain KN, Khan MA, Amin SM, Khan MS. Assessment of quality of life and its determinants in type-2 diabetes patients using the WHOQOL-BREF instrument in Bangladesh. BMC endocrine disorders. 2022 Jun 18;22(1):162.

Islam FM. Gender difference in domain-specific quality of life measured by modified WHOQoL-BREF questionnaire and their associated factors among older adults in a rural district in Bangladesh. PloS one. 2025 Jan 7;20(1):e0317113.

6. For the regression analysis, assumption checks (and the results) need to be stated.

Thank you for your valuable suggestion. We have now updated the Data Analysis section (Section 2.5) of the manuscript to include information on the assumption checks conducted prior to regression analysis. Specifically, we assessed multicollinearity, independence of residuals, and model fit to ensure the validity of the logistic regression results. The methods and outcomes of these checks have been added to ensure transparency and analytical rigor. The following paragraph are added (Line 253-260)

Before performing binary logistic regression, relevant assumptions were checked. Multicollinearity was evaluated using Variance Inflation Factor (VIF), and all predictors showed VIF values below 2.0, indicating no multicollinearity. Independence of residuals was examined using the Durbin-Watson statistic, which fell within the acceptable range of 1.6 to 2.2. Additionally, the goodness-of-fit of the regression models was evaluated using the Hosmer–Lemeshow test, with p-values greater than 0.05 suggesting a good model fit. These checks confirmed the suitability of the data for regression analysis.

7. The Discussion need to be revamped following a clearer research problem statement (not necessarily 'research gap').

Thank you for your thoughtful feedback. To address this, we revised the beginning of the Discussion section to include a clear restatement of the research problem that guided our study. We clarified that the study aimed to examine how hypertension affects the quality of life in the sociocultural and post-pandemic context of Bangladesh—particularly given the country’s unique challenges related to healthcare access, economic inequality, and COVID-19 exposure. This framing better situates the findings within the broader public health context and highlights the study’s purpose beyond simply filling a literature gap. The following paragraph are added (Line 352-360)-

“This study was driven by the need to understand how hypertension—one of the most common chronic conditions in Bangladesh—affects the quality of life of patients in the post-COVID-19 context. While the health risks of hypertension are well recognized, less is known about how it impacts daily life, particularly when combined with socioeconomic challenges and recent experiences with COVID-19. In Bangladesh, where access to healthcare is limited and economic inequalities are widespread, these factors can significantly affect both disease management and overall well-being. This study explored these issues using a multidomain approach to help inform culturally and contextually appropriate strategies that go beyond clinical care”.

8. The focus on patients with hypertension seem to be diluted given the findings on COVID-19 and diabetes status of the patients. Any conclusion drawn from the study cannot be stated as representing patients with hypertension. The manuscript title is therefore not reflecting the findings. While the manuscript does not show any fatal flaw, I find it lacking novelty that warrant a publication in a journal. It works well as a routine contemporary updates for Bangladeshi market.

We sincerely thank you for the thoughtful feedback. However, we respectfully disagree with the assertion that the focus on hypertensive patients has been diluted. The manuscript specifically recruited participants with a confirmed diagnosis of hypertension, and the primary objective was to assess their quality of life (QOL) using the WHOQOL-BREF instrument. Comorbidities such as diabetes and history of COVID-19 were analyzed not as focal populations but as covariates, reflecting real-world complexities in hypertensive care. These factors were included to examine their modifying effects on QOL outcomes among individuals with hypertension—not to shift the study's central focus.

This analytic strategy enhances the ecological validity of the findings and acknowledges the multifactorial burden hypertensive patients face, particularly in a post-pandemic Bangladeshi context. Our title clearly reflects this focus: “Quality of Life in hypertensive patients…”—emphasizing both the target population and the contextual setting.

Regarding novelty, our study adds unique value by: Applying WHOQOL-BREF among hypertensive patients in Bangladesh—a context where this instrument has been underutilized for this population; Capturing QOL after the COVID-19 pandemic, when hypertensive patients may have faced intensified health and psychosocial burdens; Identifying income, age, COVID-19 history, and lifestyle as significant determinants, which can guide locally tailored interventions. Our approach extends existing literature by using a culturally validated, multidimensional tool and by highlighting socioeconomic and pandemic-related contributors to poor QOL—an area that remains underexplored in low- and middle-income countries.

**Reviewer #2:** The manuscript reads well. Please consider the following minor comments when preparing the revised manuscript.

Typos

"...DOM1 and DOM1 were significantly (p=0.01) associated with their age, education, family type, monthly..."

Thank you for your kind response. We addressed this concern in the manuscript in the following way (Line 310-311):

"For instance, it was observed that scores in DOM1 and DOM2 were significantly (p=0.01) associated with their age, education, family type, monthly family income, status of DM, history of COVID-19, and lifestyle status of the respondents."

Methods

1. "...the study was conducted at a randomly selected hospital (Square Hospitals

Limited) from the top 10 hospitals..." Could you please identify the other nine private hospitals included in the study? What criteria were used to rank these hospitals, and what was the rationale for this ranking methodology? Furthermore, what were the reasons for focusing on private rather than public hospitals in this research? Given that private healthcare in Bangladesh can be expensive and often requires insurance, how does the study account for potential biases in representing higher-income populations?

Thank you for your insightful comment. To better capture we revised the methodology section and following paragraph is added (Line144-150)

“These hospitals were ranked based on publicly available data from health service directories and included Square Hospitals Ltd., United Hospital Ltd., Evercare Hospital Dhaka, Apollo Imperial Hospitals, Labaid Specialized Hospital, Islami Bank Central Hospital, Central Hospital Ltd., Green Life Medical College Hospital, Popular Medical College Hospital, and Asgar Ali Hospital. The ranking considered criteria such as accreditation, service quality, cardiac specialization, patient satisfaction, and availability of multidisciplinary units. Square Hospitals Ltd. was chosen through simple random sampling from this list”.

Also, following paragraph is added in method section (Line159-163)

“The decision to focus on private hospitals was based on logistical and quality assurance factors: private hospitals in Dhaka maintain more consistent electronic health records, have established non-communicable disease programs, and allow for standardized patient follow-up. Our study does include socioeconomic diversity—over 65% of participants reported a monthly income of ≤ USD 853.14, suggesting the inclusion of middle- and lower-income urban patients—not solely affluent individuals. Thus, it is allowing for partial mitigation of income-related selection bias”.

2. how authors come up with USD853.14 as good or bad household income.

Thank you for your thoughtful query. The threshold of USD 853.14 was derived from the median monthly household income reported by our study participants, which was approximately 90,000 BDT. This was converted to USD using the prevailing exchange rate at the time of data analysis (1 USD ≈ 105.45 BDT). We used the median as a cut-off to stratify participants into lower- and higher-income groups, a standard approach in health research when assessing socioeconomic disparities within a study population. This method allowed us to examine how income variation—relative to the sample’s central tendency—impacted quality of life (QOL) among hypertensive patients. While this threshold does not represent the national poverty line, it serves as a contextually valid measure for income differentiation within our study population.

3. "...A random sampling technique was employed in this study to ensure the generalizability of the findings." The total number of eligible patients from which the random sample of 300 was drawn. So. the initial patient pool is missing from that statement alone.

Thank you for this insightful observation. To address your concern, we have now clarified the source population size in the Methods section. During the data collection period, a total of 600 eligible hypertensive patients were listed in the admission logs across the two departments (Cardiac & Vascular Surgery and Cardiology) of Square Hospitals Limited. From this pool, a systematic random sampling technique was used to select every second eligible patient, resulting in a final sample of 300 participants. This detail has been added to ensure transparency and improve the reproducibility of our sampling approach. We have revised our methodology and this would be reflected in the manuscript line 152-155.

4. "...The overall prevalence of hypertensive patients in the Dhaka reflected 31%..."

The observed hypertension prevalence of 31% in Dhaka suggests that odds ratios from logistic regression may not be a good approximation of prevalence ratios. Could you elaborate on why logistic regression was chosen over methods known to directly estimate prevalence ratios, such as Poisson regression with robust standard errors?

Thank you for this thoughtful observation. We would like to clarify that our study was not designed to estimate the prevalence of hypertension in the general population. Rather, it focused exclusively on patients already diagnosed with hypertension to examine associations between their quality of life (QOL) and various sociodemographic, clinical, and lifestyle factors. However, in our study, logistic regression was considered appropriate for the following reasons:

First, the primary objective was not to estimate the prevalence of hypertension—since all participants were already hypertensive—but to explore factors associated with dichotomized quality of life (QOL) outcomes (i.e., good vs. poor) across the WHOQOL-BREF domains. Each QOL domain was treated as a binary dependent variable, making logistic regression a suitable and commonly used approach in such contexts.

Second, logistic regression allows for robust modeling of the relationship between categorical outcomes and multiple predictors, which is well-supported in the literature assessing QOL among patients with chronic conditions.

Third, model assumptions were rigorously checked. As noted in the revised manuscript (Lines 253–259), we verified multicollinearity using VIF, assessed residual independence with the Durbin-Watson test, and confirmed model fit with the Hosmer–Lemeshow test—all of which met acceptable thresholds.

We acknowledge that Poisson regression with robust standard errors is a valid alternative for estimating prevalence ratios and will consider this in future analyses where the outcome of interest is prevalence-based rather than association-based.

5. Include strength and weakness of the study in discussion section

Thank you for your thoughtful suggestion. We have revised the final paragraphs of the discussion section to include both the strengths and limitations of our study (lines 421-452)

Several limitations should be acknowledged. First, cross-sectional design restricts our ability to infer causality. Second, as the study was conducted in a private tertiary hospital in urban Dhaka, the findings may not be fully generalizable to hypertensive populations in rural or underserved areas. The reliance on self-reported data also introduces the potential for reporting bias. Additionally, due to the absence of local data on binary QOL outcomes, we approximated the sample size using an average WHOQOL-BREF domain score (0.76) drawn from published literature. While this approach was practical, it remains an approximation. Future studies would benefit from pilot data or the use of stratified QOL thresholds to support more precise, context-specific sample size calculations. Despite these limitations, the study has several strengths. It was conducted in a well-resourced tertiary care setting that offers structured follow-up and equitable access to services for both men and women. This may have mitigated gender-related disparities in care and health outcomes often seen in less-structured, community-based settings. Although urban healthcare facilities can limit generalizability, the consistency of care and documentation in such settings reduced variability and enhanced the reliability of our findings. As private hospitals become increasingly integrated into Bangladesh’s national NCD care framework, understanding patient outcomes in these settings is both timely and policy relevant. Rather than constraining generalizability, the study highlights key determinants of QOL—such as age, diabetes, income, and COVID-19 history—that are likely to be relevant across diverse healthcare contexts. We also employed a validated multidimensional tool (WHOQOL-BREF), used systematic random sampling, and included participants from varied sociodemographic backgrounds, all of which enhanced the internal validity of the study. Furthermore, the use of Spearman’s correlation and regression analyses allowed for a robust examination of factors associated with QOL. Importantly, this study adds new insights to the existing literature by exploring the impact of COVID-19 exposure on the quality of life of hypertensive patients. Here, the “post-pandemic” period refers to March–June 2023—a time when Bangladesh had exited the acute phase of the pandemic, but many individuals, especially those with chronic conditions, continued to face challenges such as delayed care, financial strain, and psychological stress. This period represents a distinct context from both the peak-pandemic and pre-pandemic phases and offers meaningful insights into how lingering challenges impact the quality of life in hypertensive patients. By examining this context, the study sheds light on the lived experiences of a population whose quality of life—impacted by both chronic illness and pandemic-related stressors—has been largely underexplored in current literature.

**Reviewer #3:** The manuscript under review offers a reasonably thorough examination of public health patterns, supported by an adequate sample size (n=300), which meets the conventional minimum threshold for statistical significance. The authors' application of statistical methods throughout the paper is generally appropriate (lines 24–28), though there are persistent issues with terminology usage—most notably the incorrect treatment of plural forms such as "data" (line 22) and "statistics" (lines 24, 28), which detract from the academic rigor.

We appreciate your attention to academic rigor and clarity. In response, we carefully reviewed the manuscript and revised the sentences in the following:

- The term “data”, which is plural, has been corrected to be used with plural verb forms where necessary. For example, the sentence originally written as “The data was collected…” has been revised to “The data were collected…” (Line 22-23).
- Similarly, “statistics”, when referring to numerical data (plural), has been adjusted accordingly. For instance, we have changed “Descriptive statistics was used…” to “Descriptive statistics were used…” (line 24-25).

Early in the manuscript, clarity in definitions would benefit the general readership. For instance, the description in line 43 should be revised to read: “Hypertension, commonly referred to as high blood pressure,” as “hypertension” is the correct clinical term.

Thank you. We agree that precise definitions are essential, particularly for general readership. In response, we have revised the sentence to read (line 43)-

“Hypertension, commonly referred to as high blood pressure, is one of the leading causes of death among non-communicable diseases worldwide.”

There are more substantive concerns regarding methodological transparency. On line 119, the claim that Dhaka was selected through “multi-stage random sampling” raises doubts, particularly when the authors themselves cite the city’s extensive healthcare infrastructure as a benefit. This contradiction suggests convenience sampling may have played a role, undermining the generalizability of the findings.

Thank you, your feedback is justified indeed. I have updated the sentence with more clarity. The selection of Dhaka as the study site was part of a multistage random sampling strategy designed to ensure methodological rigor and geographical representation within resource constraints. In the first stage, the Dhaka division was randomly selected from among the eight administrative divisions of Bangladesh. This randomization was essential to avoid selection bias at the macro-geographic level. In the second stage, Dhaka city was included as it falls within the randomly chosen division, and offers a dense population and a diverse patient base, both of which are critical for studying quality of life among hypertensive individuals. In the third stage, Square Hospitals Limited was randomly selected from a list of top tertiary care hospitals in Dhaka that have specialized cardiac and hypertension services.

While Dhaka’s advanced healthcare infrastructure is recognized as a benefit, it was not the primary criterion for site selection—rather, it was a practical advantage of the hospital that was randomly chosen. The hospital-level randomization further supports the robustness of the sampling framework. Therefore, despite the inherent strengths of Dhaka’s health system, the use of multistage random sampling remains valid, and steps were taken at each stage to minimize bias and enhance the generalizability of findings within similar urban healthcare settings in Bangladesh.

Similarly, the justification provided for the sample size (line 126) lacks credibility. While the authors imply that their choice of 300 participants is a deliberate methodological decision, it more likely reflects the standard threshold for statistical relevance. Further clarification is needed to distinguish between statistical necessity and genuine methodological reasoning.

Certainly, we acknowledge the query. However, the sample size of 300 participants was determined using a standard statistical formula n=Z^2^pq/d^2^, where:

- Z = 1.96 (for 95% confidence),
- p = 0.76 (based on prior QOL domain averages in hypertensive populations),
- d = 0.05 (margin of error).

This yielded a base sample size of 280. To account for non-response and potential data entry errors, we added 7%, bringing the total to 300. This number is not arbitrary, nor merely a standard threshold, but the result of a quantifiable and transparent calculation tailored to the study’s objectives. Moreover, a sample size of 300 enhances the statistical power for subgroup analysis across multiple QOL domains, supports the generalizability within an urban hypertensive population, and aligns with best practices in cross-sectional studies using WHOQOL-BREF. Therefore, both statistical necessity and methodological rigor informed the chosen sample size.

The paper mentions that the data collection instrument was “pre-tested” (line 133), but fails to specify critical details—namely, the demographic or size of the test group. It is unclear whether the pre-test was conducted among the study’s target population or a separate group, which raises questions about the reliability and validity of the questionnaire.

We have critically revised this section in response to your valuable feedback. The purpose of the pre-test was to ensure that the questionnaire was culturally appropriate, clearly understood, and contextually relevant for the target population. Based on feedback from the pre-test, minor revisions were made to improve language clarity and flow. This process helped to enhance the face validity and content validity of the instrument in the local context.The following has been revised to the manuscript in the Methods section (Lines 175–181):

“Prior to formal data collection, a pre-test was conducted with 15 hypertensive patients who were demographically similar to the study population but not included in the final sample. The aim was to assess the clarity, cultural relevance, and contextual appropriateness of the Bengali-translated WHOQOL-BREF. Minor modifications were made based on participant feedback to improve wording and flow, thereby strengthening the instrument’s face and content validity.”

On a conceptual level, the discussion around COVID-19 exposure (lines 195–196) appears disjointed. The finding that 59.7% of participants had been exposed to COVID-19 is not inherently surprising and lacks a compelling rationale or comparison benchmark that would make this statistic meaningful.

Thank you for your valuable comment. We have revised the discussion to clarify that the 59.7% COVID-19 exposure rate among hypertensive patients is not intended to be a surprising statistic, but rather serves as an important contextual variable. The study was conducted during the post-pandemic period (March–June 2023), a time when the lasting effects of the pandemic—such as delayed medical care, financial hardship, and psychological stress—continued to affect patient well-being.

In our analysis, COVID-19 history was included as a covariate to evaluate its impact on quality of life (QOL), rather than as a primary outcome. Regression findings showed that prior COVID-19 infection was significantly associated with lower QOL scores across multiple domains, emphasizing its role in exacerbating the burden of hypertension.

These revisions improve conceptual clarity and reinforce the relevance of COVID-19 exposure as a critical contextual factor within the broader framework of QOL assessment in post-pandemic, resource-constrained settings like Bangladesh.

In terms of structure, there is some redundancy toward the end of the manuscript (lines 321–327), where information is repeated unnecessarily. Additionally, the paper concludes with a statement of limitations (line 329), which, while important, may not be the most impactful way to close the manuscript. A more compelling conclusion could emphasize the broader implications or recommendations, while still incorporating the study’s limitations.

Thank you for this thoughtful and constructive suggestion. In response, we have carefully revised the manuscript to remove the redundancy by merging repetitive points and improving sentence flow.

While we briefly mention the study’s limitations, we now focus on the broader implications of the findings. The revised conclusion (line 455-463) emphasizes how factors such as age, diabetes, COVID-19 history, and income can inform culturally appropriate, patient-centered interventions. We also include practical recommendations for healthcare providers and policymakers, especially in post-pandemic and resource-limited settings like Bangladesh.

A final critical consideration is whether the paper offers culturally appropriate strategies, as alluded to earlier in the text (line 85). This is a potentially valuable contribution, yet it remains unclear whether such strategies are proposed or substantiated in the discussion.

Thank you. Such concept is now discussed in the last para of the discussion section.

In summary, the manuscript provides a solid foundation in terms of data collection and statistical analysis but is weakened by inconsistencies in methodological transparency, terminological imprecision, and rhetorical structure. Overall, the study offers a meaningful contribution to multiple disciplines and thus has inherent value to the research community writ large.

We sincerely appreciate your comment recognizing our study’s contribution. We have carefully reviewed the manuscript in light of the concerns raised regarding methodological transparency, terminological precision, and rhetorical structure. Substantive revisions have been made throughout the manuscript to address these issues. Specifically, we have clarified the sampling strategy, elaborated on the pre-testing process, strengthened the justification for the sample size, and revised key sections for consistency and clarity. We also refined the conclusion to better reflect the study’s broader implications while maintaining a concise discussion of limitations. We believe these improvements enhance both the scientific rigor and readability of the manuscript, and we are grateful for the reviewer’s guidance in this process.

**Reviewer #4:** This manuscript investigates the quality of life (QOL) among hypertensive patients in a selected tertiary hospital in Dhaka, Bangladesh, using the WHOQOL-BREF instrument.

While the study addresses an interesting public health question, it suffers from multiple fundamental flaws related to the representativeness of the study population. Due to these issues in the selection of a highly specific and unrepresentative study site (Square Hospitals Limited), which severely compromises the generalizability and external validity of the findings for the broader hypertensive population in Dhaka, or Bangladesh, this manuscript is not suitable for publication. The conclusions drawn cannot reliably inform public health strategies for the majority of the population. Combined with other methodological and interpretative concerns, these issues warrant Rejection.

A. Fundamental Issues:

1. The study was conducted at Square Hospitals Limited, described as a "leading tertiary care facility" and one of the "top 10 hospitals" in Dhaka with a dedicated cardiac department. Such premier institutions in Dhaka are only accessible primarily to a more affluent segment of the population. This deliberate selection of a high-end, private hospital introduces a significant selection bias. The patient population from such a facility is unlikely to be representative of the general hypertensive population in Dhaka, and certainly not of Bangladesh, where a substantial portion of the populace is underprivileged and relies on public healthcare facilities or less expensive private options.

Thank you for your thoughtful feedback. We respectfully acknowledge the concern regarding the representativeness of the study population due to the selection of Square Hospitals Limited, a high-end private tertiary care center. We would like to clarify and justify this methodological choice while acknowledging its limitations.

Our primary aim was to assess the quality of life (QOL) among hypertensive patients using the WHOQOL-BREF instrument within a structured, reliable clinical setting that ensured accurate patient history, consistent diagnosis, and completeness of records. The hospital considered criteria such as accreditation, service quality, cardiac specialization, patient satisfaction, and availability of multidisciplinary units. Square Hospitals Ltd. was chosen through simple random sampling from this list. The decision to focus on private hospitals was based on logistical and quality assurance factors: private hospitals in Dhaka maintain more consistent electronic health records, have established non-communicable disease programs, and allow for standardized patient follow-up (reflected in line 148-162).

We agree that patients at private hospitals often represent a higher socioeconomic bracket. However, the study explicitly acknowledges this limitation in the discussion section, stating that “focusing on a private facility could overrepresent middle- to higher-income groups, and that findings may restrict the generalizability of the findings to the broader hypertensive population in Bangladesh, particularly those living in rural or underserved areas (line 430-438). Although urban healthcare facilities can limit generalizability, the consistency of care and documentation in such settings reduced variability and enhanced the reliability of our findings (line 430-443).

Despite this limitation, we believe the findings offer valuable insight into the existing body of hypertension research by examining how COVID-19 exposure affects quality of life (QOL). It sheds light on the lived experiences of a segment of the population whose QOL—shaped by both chronic conditions like hypertension and pandemic-related stressors—has remained underexplored in current literature.

2. Consequently, the findings regarding QOL and its determinants from this specific, potentially socioeconomically advantaged, patient cohort cannot be generalized to the broader population of hypertensive patients in Bangladesh. The study's conclusions and recommendations, if based on this unrepresentative sample, may be misleading for public health planning and interventions aimed at the general population. The manuscript aims to provide insights for "Bangladesh" (Abstract, Background, Conclusion), but the sampling strategy fundamentally undermines this goal.

We sincerely appreciate your thoughtful feedback on the issue of representativeness and generalizability. We fully acknowledge that conducting the study at Square Hospitals Limited—a private tertiary care facility in Dhaka—may raise concerns regarding socioeconomic and geographic inclusivity.

However, several steps were taken to address potential bias and enhance the applicability of the findings:

a. Sampling Strategy: Although the study was conducted in a private facility, a *systematic random sampling* approach was employed, and the study included participants with varied sociodemographic backgrounds. Notably, 65.3% of respondents had a monthly family income of ≤ USD 853.14, indicating representation of economically diverse groups, not only the affluent.
b. Rationale for Site Selection: The selection of Square Hospitals was based on its comprehensive electronic medical records, consistent chronic disease follow-up systems, and logistical feasibility for standardized data collection—factors critical for methodological rigor. Square Hospitals were *randomly selected from the top ten cardiac-focused tertiary hospitals in Dhaka*, using a transparent and documented selection process.
c. Clarification of Scope: While we acknowledge that the study was conducted in a single urban private facility, we respectfully clarify that the objective was to assess quality of life (QOL) within a structured, resource-adequate setting where chronic disease management is well established. Although urban care facilities restrict generalizability, this environment enabled consistent data collection and reduced variability stemming from issues like inadequate follow-up or incomplete documentation—common challenges in lower-tier facilities. These focused insights are valuable for understanding how hypertension affects QOL among populations with regular access to care, an increasingly important segment Bangladesh’s evolving healthcare landscape.
d. Strengthening Relevance for Public Health: While we appreciate the reviewer’s concern, we believe that findings from structured tertiary care settings like Square Hospitals still hold strong public health value. These settings offer critical benchmarks for assessing the quality of life among hypertensive patients, especially in urban areas where a growing number of individuals seek care. As private hospitals are increasingly integrated into Bangladesh’s national NCD care system, capturing patient outcomes in these environments is both timely and policy relevant. Rather than limiting generalizability, our study highlights key determinants of QOL—such as age, diabetes, income, and COVID-19 history—that are likely to be influential across diverse healthcare settings. Thus, our findings offer actionable insights that can inform scalable, context-sensitive models of care.

3. The manuscript does not adequately acknowledge or discuss the profound limitations imposed by this choice of study site on the external validity of its findings. While the discussion mentions "focusing on specific region (Dhaka) may limit the generalizability," this does not capture the critical issue of socioeconomic selectivity within Dhaka due to the choice of hospital. Furthermore, the authors’ interpretation in multiple instances appear quite out of scope.

Thank you for this important observation. We acknowledge that conducting the study in a private tertiary care hospital in Dhaka may limit the external validity of the findings, particularly for rural or underserved populations. In response, we have revised the Discussion and Conclusion sections to more clearly state this limitation and to avoid overstating the scope of generalization.

However, we respectfully note that our study does include socioeconomic diversity—over 65% of participants reported a monthly income of ≤ USD 853.14, suggesting the inclusion of middle- and lower-income urban patients—not solely affluent individuals. Square Hospitals was selected due to its structured chronic disease follow-up and availability of reliable records, making it well-suited for examining QOL outcomes in a controlled, urban healthcare context.

Our interpretations have been reviewed and adjusted to ensure alignment with the study’s scope. The revised manuscript now clearly states that findings are most applicable to urban, structured care settings Rather than generalizing to all population of Bangladesh. and are not intended to generalize to all hypertensive populations in Bangladesh. We believe these insights still contribute valuably to public health planning by offering reference points for quality of life among hypertensive patients in similar healthcare contexts.

B. Rationale and Contextualization

1. While the study mentions the COVID-19 pandemic's impact, the "post-pandemic" aspect could be more clearly defined and integrated. It's unclear what specific timeframe "post-pandemic" refers to in this study and how this phase distinctly influences QOL compared to the pandemic phase or pre-pandemic times.

Thank you for your valuable feedback. For addressing this concern, we have revised the discussion section (Line 443-452)

To clarify, the term “post-pandemic” in this study refers to the period between March and June 2023, when COVID-19-related restrictions and emergency responses had subsided in Bangladesh, but residual impacts on healthcare systems and psychosocial well-being remained. Although infection rates were stable during this time, the lasting effects of the pandemic—including economic disruptions, deferred chronic disease management, and elevated stress—continued to influence quality of life among hypertensive patients. This transitional phase is distinct from both the acute pandemic period and the pre-pandemic baseline, offering a unique opportunity to assess how individuals adapt to long-term consequences in the absence of active crisis. Integrating this post-pandemic context enhances the relevance of our findings, especially in informing chronic disease care strategies in ongoing recovery phases.

2. The rationale for choosing WHOQOL-BREF over other instruments like EQ-5D (which the authors mention was used in Indonesia during the pandemic) could be strengthened, particularly in the context of previous HRQOL studies in Bangladesh.

Thank you for this thoughtful comment. We acknowledge the importance of clearly articulating our rationale for selecting the WHOQOL-BREF over other instruments such as EQ-5D. Now we clearly mention it in the introduction (Line 76-87) and discussion section (Line 312-418).

In the context of Bangladesh, the WHOQOL-BREF has been more widely used and validated in prior health-related quality of life (HRQOL) studies, including among patients with chronic conditions such as diabetes and older adults [36,37]. These studies have demonstrated strong psychometric properties, including construct validity and multidimensional applicability in the Bangladeshi context. Moreover, WHOQOL-BREF offers a broader evaluation of quality of life, encompassing four key domain-physical health, psychological well-being, social relationships, and environmental factors—which are especially relevant when assessing the complex, multifactorial burden of hypertension in the post-pandemic setting. In contrast, while EQ-5D is widely used and efficient, it offers a more limited scope and is less commonly validated for use among diverse sociodemographic groups in Bangladesh.

C. Methods

1. Patients were "randomly selected from two departments... using the patient register." Further details on the randomization process are needed. Were all patients in the register eligible? How was systematic bias avoided? Are these two departments (Cardiac & Vascular Surgery and Cardiology) representatives of all hypertensive patients, even within this hospital (e.g., what about patients managed in general medicine or outpatient clinics for hypertension without acute cardiac events)?

To minimize selection bias and ensure systematic rigor, we employed a systematic random sampling technique. Initially, 500 eligible hypertensive patients were identified from the admission registry of the Cardiac & Vascular Surgery and Cardiology (Interventional) departments at Square Hospitals Limited. Eligibility was determined based on (1) a confirmed diagnosis of hypertension for at least six months, (2) current admission under one of the two departments, (3) absence of mental or physical disability, and (4) provision of informed consent. From this eligible pool, every third patient in the admission log was selected, yielding a final sample of 300 participants.

We recognize the reviewer’s concern regarding departmental representation. We selected these two departments because they have dedicated and structured clinical pathways for managing hypertensive patients. They also regularly treat patients with comorbid cardiac conditions, which aligns with the clinical focus of our study. While general medicine and outpatient clinics also manage hypertensive patients, they were not included due to variability in case severity, inconsistent documentation, and irregular follow-up, which could compromise the uniformity and accuracy of data collection.

Importantly, our sample reflects a range of socioeconomic backgrounds—over 65% of participants reported a monthly income of ≤ USD 853.14, suggesting that the study did not solely include affluent individuals. We believe the use of systematic random sampling, clear eligibility criteria, and data collection from departments with well-established cardiac care protocols helped minimize selection bias and strengthened the internal validity of our findings.

We have revised the Methods section (Section 2.2) to further elaborate on this sampling process and rationale for departmental selection. In Section 2.2, the following is clarified (Line 152-159):

“Initially, 600 hypertensive inpatients from the Cardiac & Vascular Surgery and Cardiology (Interventional) departments were identified. Every second patient meeting the inclusion criteria was selected using systematic random sampling. These departments were chosen due to their structured, well-documented care pathways for patients with hypertension and related cardiac conditions, facilitating reliable data collection. Patients managed in outpatient or general medicine settings were excluded due to variability in hypertension management protocols and inconsistent follow-up practices.”

2. The proportion (p) used for sample size calculation was 0.76, stated as "the QOL among hypertensive patients expressed by mean score of domains ranging from 0.65 to 0.88, with 0.76 considered as the average" (citing a study from rural Vietnam). Using an average of a range of mean scores as a 'proportion' for sample size calculation for a binary outcome (implied by later categorizing QOL as 'Good/higher' vs 'Poor to moderate/lower') is methodologically questionable. The justification for p=0.76 needs to be clearer and more appropriate for the intended analysis.

We acknowledge that using *p = 0.76*—derived from the average of domain mean scores—is an nontraditional approach for calculating sample size based on a binary outcome. This value was informed by prior research (Ha et al., 2014, *BMC Public Health*), where WHOQOL-BREF domain scores among hypertensive patients ranged from 0.65 to 0.88. In the absence of post-pandemic, population-specific data on the proportion of patients with “good” QOL in Bangladesh, we used the average score (0.76) as a practical proxy to estimate the likely proportion of participants reporting higher QOL.

Although this estimate was based on continuous data, it was aligned with our later binary categorization of QOL (i.e., “Good/higher” vs. “Poor to moderate/lower”). We acknowledge this as an approximation rather than an exact measure, but it offered a feasible solution in the context of limited local data.

To ensure transparency, we have revised the Methods section to clarify this approach and have also acknowledged it as a methodological limitation in the Discussion.

In Section 2.2, we revised the paragraph as follows (Line 166-172):

“The initial sample size of 280 was calculated using the formula n = Z²pq/d², where Z = 1.96, d = 0.05, and p = 0.76. The value of p was based on the average of WHOQOL-BREF domain mean scores (range: 0.65–0.88) reported in a similar population [23], in the absence of precise binary prevalence data. Although this value originates from continuous data, it was used as a proxy to estimate the proportion of participants likely to report good quality of life. An additional 7% was added to account for potential non-response or error, resulting in a final sample size of 300.”

In the Discussion section, we added (Line 425-431):

“We acknowledge that the use of a continuous average score (0.76) to estimate a binary proportion introduces a methodological limitation. This decision was made due to a lack of local prevalence data on binary QOL outcomes among hypertensive patients in Bangladesh. Future studies may benefit from pilot estimates or stratified QOL thresholds to inform more precise sample size determination.”

3. The manuscript states that poor lifestyle was "measured considering the variables like physical activity, sleeping pattern, deleterious habit etc." (Results, Table 2 footnote). This is vague. The specific components, how they were measured, and how they were combined to classify "poor lifestyle" need to be explicitly detailed in the methods.

Thank you for this insightful comment. We agree that further clarification is warranted regarding the operationalization of the "poor lifestyle" variable. In response, we have revised the Methods section (specifically under subsection 2.4 “Questionnaire Design” and 2.5 “Data Analysis”) to provide greater transparency on how lifestyle was assessed and categorized.

Specifically, "lifestyle" was evaluated through three components:

a. Food habit: Several types of food intake (healthy and unhealthy food) including amount of salt intake per day by the participants and their meal frequency were considered to assess their healthy or unhealthy dietary practice as part of their lifestyle pattern. More than 1 tea spoon salt intake was considered as unhealthy practice. In addition, alcohol intake was considered as unhealthy habit.
b. Physical activity: Participants reporting at least 30 minutes per day or 150 minutes per week of moderate-intensity activity (e.g., walking) were categorized as physically active, based on WHO recommendations. Those reporting less than this threshold were classified as physically inactive or below standard practice.
c. Sleeping pattern: Adequate sleep was defined as 6–8 hours (Standard sleeping) of nightly sleep without regular complaints of insomnia or frequent no daytime napping. Respondents reporting less than 6 (below standard) or more than 8 hours of sleep (Oversleeping), or those with self-reported insomnia or frequent no daytime napping, were considered to have a poor sleep pattern.
d. Perception of stress and anxiety: This included perception about stressed and anxious feeling. Participants who felt stressed and anxious were classified as having unhealthy lifestyle.

Each of these components was scored as binary (0 = unhealthy; 1 = healthy). A cumulative score ranging from 0 to 13 was generated for each participant and then converted in percentage score. Those scoring ≤62% (mean 61.56) were classified as having a “poor lifestyle”, while those scoring >62% categorized as having a “moderate/healthy lifestyle”. This scoring approach was informed by previous studies assessing lifestyle risk aggregation in relation to chronic disease outcomes.

4. The scores for QOL domains were dichotomized using 50% as a cutoff ("Scores above 50% were considered as 'Good/higher QOL'; while below 50% as 'Poor to moderate/lower QOL'"). This transformation of a continuous scale into a binary variable can lead to loss of information. Justification for this specific cutoff and the potential impact of this dichotomization should be discussed.

Thank you for your valuable feedback. We acknowledge that converting the continuous WHOQOL-BREF domain scores into binary categories using a 50% cutoff may lead to some loss of information. However, we chose this approach for two main reasons. First, the 50% threshold has been commonly used in previous studies to help interpret results more clearly and to distinguish between "poor to moderate" and "good" quality of life in public health contexts (e.g., Adamu et al., 2022; Ha et al., 2014; Skevington & McCrate, 2012). This binary classification made it easier to identify individuals who might benefit most from interventions, which can support more practical public health planning.

Second, in our specific context, dichotomization allowed for a clearer stratification of the hypertensive population when exploring associations with sociodemographic and clinical factors using logistic regression. While we understand this approach may lead to some loss of detailed information, our main objective was to present the data in a way that supports meaningful, actionable insights—particularly in a resource-limited setting like Bangladesh.

We have acknowledged this limitation in the revised manuscript and clarified in both the Methods and Discussion sections that the 50% cutoff was selected based on previous literature and to enhance the interpretability and comparability of our findings.

5. Statistical Analysis: The use of backward elimination for regression analysis can be problematic as it may lead to model instability and exclusion of important confounders based on statistical significance rather than theoretical importance.

Thank you for your insightful comment. We understand that using backward elimination in regression analysis can have some drawbacks, such as the possibility of removing important confounding variables. However, in our study, we selected the initial variables based on both theoretical relevance and findings from existing literature.

We used backward elimination mainly to simplify the model and avoid overfitting, considering our sample size. This method helped us identify the most important factors associated with quality of life in a clear and manageable way. We also checked for multicollinearity (VIF < 2) and confirmed the model's goodness-of-fit using the Hosmer–Lemeshow test.

We have clarified this in the revised manuscript.

D. Interpretation and Discussion

1. Given the significant limitation of the study site, any conclusions drawn about the QOL of hypertensive patients in Bangladesh are likely overstated. The discussion should be heavily tempered by this limitation.

Thank you for your thoughtful comment. We agree that the single-site nature of our study is a limitation and may limit the generalizability of the findings to the broader population of hypertensive patients in Bangladesh. However, we would like to emphasize that our study site large tertiary care hospital—serves a diverse group of patients from various socioeconomic backgrounds, both urban and peri-urban. As such, it provides valuable insights into the lived experiences and quality of life challenges faced by hypertensive patients in a real-world clinical setting.

Rather than overstating the findings, our intention is to highlight key associations that can inform future research and policy discussions. We have now further clarified this point in the Discussion section and explicitly acknowledged that our conclusions should be interpreted within the context of the study setting. At the same time, we believe that our findings offer a meaningful contribution to the limited body of research on QOL among hypertensive patients in Bangladesh.

2. When comparing findings (e.g., gender not being significant vs. an Ethiopian study where it was), the discussion should consider that the highly selective nature of the current study's sample might explain these discrepancies, rather than just "cultural or regional differences."

Thank you for your valuable feedback. We agree that differences in findings, such as the non-significant association between gender and QOL in our study compared to the Ethiopian study, may be influenced by the nature of the study sample. Our study was conducted in a large tertiary care hospital that provides structured follow-up care and relatively equal access to services for both male and female hypertensive patients. This healthcare context may have minimized gender-related disparities in health status, health-seeking behavior, and access to resources, which are often more pronounced in community or rural settings.

We believe this is a strength of our study rather than a limitation. It highlights how access to consistent care in an urban clinical setting may help reduce QOL differences across gender. In contrast, the Ethiopian study was based on a broader community sample where such disparities may remain unaddressed. Therefore, the absence of a significant gender effect in our findings reflects the influence of improved healthcare access rather than sample bias.

We have now revised the Discussion section to incorporate this explanation, emphasizing that the context of our sample provides a valuable perspective on how health system structure can influence QOL outcomes.

3. The study found lower monthly family income was associated with poor QOL. In a sample drawn from a high-end hospital, this "lower income" group is still relatively well-off compared to the general population, making the interpretation of this finding misleading.

We respectfully disagree with the implication that the association between lower monthly family income and poor quality of life (QOL) is misleading due to the high-end nature of the study setting. Although Square Hospital is a tertiary-level private facility, our sample demonstrated considerable socioeconomic variation. Notably, more than 65% of participants reported a monthly family income of USD ≤853.14—an income level that falls within the lower-middle bracket in urban Bangladesh. This indicates that our study population was not limited to affluent individuals but included a diverse economic cross-section.

Importantly, our findings are consistent with existing evidence showing that even in relatively well-resourced urban settings, individuals with lower income often face worse health outcomes and diminished QOL. The strong and significant association observed between lower income and poor QOL—particularly in the environmental domain (AOR = 16.89, *p* = 0.01)—highlights how socioeconomic disadvantages continue to impact patient well-being, regardless of the care environment.

Rather than being misleading, this association reflects real and persistent intra-urban disparities. It emphasizes the need for income-sensitive approaches to hypertension care, even within structured, private healthcare settings. Our findings contribute to a more nuanced understanding of how economic vulnerability influences health, reinforcing the importance of inclusive public health strategies that address the needs of all income groups.
