# Supplementary material for: Quality of life in hypertensive patients using the WHOQOL-BREF instrument in the post-pandemic Bangladesh: A cross-sectional study
Source: PLoS One. 2026 Jan 16;21(1):e0340897. doi: 10.1371/journal.pone.0340897 (PMC12810801; doi:10.1371/journal.pone.0340897)
Supplement: Attachment — Submitted filename: Reviewer Response_Doc_26_7_25.docx [file pone.0340897.s007.docx]

Thank you for the revision request and considering our article for second revision. We are mentioning our revisions that made according to the recommendations. The point by point reply is mentioned below and revised manuscript is enclosed in the submission engine.

Journal Requirements:

1. If the reviewer comments include a recommendation to cite specific previously published works, please review and evaluate these publications to determine whether they are relevant and should be cited. There is no requirement to cite these works unless the editor has indicated otherwise. 
Thank you for the suggestion. We carefully reviewed all prior queries recommended by the reviewer. Each of the suggested references was assessed for its relevance, methodological alignment, and contextual fit with the current study, which focuses on evaluating quality of life (QOL) among hypertensive patients in post-pandemic Bangladesh using the WHOQOL-BREF instrument.

- Relevant citations that aligned with our research objectives and added value to the background or discussion have been incorporated into the revised manuscript.
- Irrelevant or tangential references, those that did not directly support our study objectives, population, or methodological framework—were respectfully excluded, in accordance with the editorial guideline.

After a rigorous revision we updated our manuscript again. All incorporated references have been clearly cited in the appropriate sections of the manuscript and added to the reference list. We have ensured that only meaningful and scientifically relevant citations are included to maintain the coherence and focus of the manuscript.

Thank you for encouraging us to strengthen the manuscript through careful evaluation of the literature.

2. Please review your reference list to ensure that it is complete and correct. If you have cited papers that have been retracted, please include the rationale for doing so in the manuscript text, or remove these references and replace them with relevant current references. Any changes to the reference list should be mentioned in the rebuttal letter that accompanies your revised manuscript. If you need to cite a retracted article, indicate the article’s retracted status in the References list and also include a citation and full reference for the retraction notice.

We sincerely appreciate the reviewer’s suggestion regarding the reference list. In response, we conducted a comprehensive review of all citations included in the manuscript titled *“Quality of Life in Hypertensive Patients Using the WHOQOL-BREF Instrument in Post-Pandemic Bangladesh: A Cross-Sectional Study Followed Analytical Approach.”*

- No retracted articles were identified among the cited references. We verified the status of each reference using reliable sources, including journal websites and the Retraction Watch database.
- All references have been reviewed and updated for accuracy and completeness, including author names, publication years, titles, journal names, volume/issues, and DOIs (where applicable).
- We also ensured that the referenced literature aligns with the scope, objectives, and context of our study, particularly regarding the use of WHOQOL-BREF, hypertension-related QOL assessments, and post-pandemic public health research in LMIC settings like Bangladesh.

The revised reference list reflects these updates, and the manuscript has been corrected accordingly. These changes have also been documented in the tracked version of the manuscript.

Thank you for your valuable feedback in ensuring the rigor and integrity of our work.

Additional Editor Comments:
Thanks for your response according to the reviewers comments. Please properly revise the English and format and submit within Sep 08 2025 11:59PM.

Thank you for your guidance. In response to your editorial note:

- We have conducted a thorough revision of the manuscript’s language to ensure clarity, grammatical accuracy, and professional tone throughout the text.
- The formatting has been carefully reviewed and corrected to align with the journal’s submission requirements, including headings, tables, figure legends, citations, and reference formatting.
- All changes have been tracked in the revised manuscript for transparency.

We appreciate your feedback and continued support throughout the review process.
