# Supplementary material for: Quality of life in hypertensive patients using the WHOQOL-BREF instrument in the post-pandemic Bangladesh: A cross-sectional study
Source: PLoS One. 2026 Jan 16;21(1):e0340897. doi: 10.1371/journal.pone.0340897 (PMC12810801; doi:10.1371/journal.pone.0340897)
Supplement: Attachment — Submitted filename: PONE-D-25-12356 (11.25).docx [file pone.0340897.s008.docx]

Dr. Nasrin Akter and colleagues investigated Quality of Life in hypertensive patients using the WHOQOL-BREF instrument in Bangladesh. The major finding is that hypertensive patients have low QOL in psychological and social domains, with specific factors influencing QOL across all domains.

1. It is recommended that the title be revised to “Quality of Life in hypertensive patients using the WHOQOL-BREF instrument in the post-pandemic Bangladesh: A cross-sectional study”.
2. References 1 and 2 are listed in reverse order, as are References 24 and 25. Please revise these.
3. Line 212 states "Those with frequent no daytime napping were considered to have a poor sleep pattern." Are there any relevant references to support this argument?
4. As mentioned in Line 237, the "thirteen-item index" lacks one item in its parenthetical explanation. Please supplement it.
5. Table 1 and Table 2 are referenced in reverse order: Table 2 is mentioned in Line 279 while Table 1 is referenced in Line 297. Please correct these.
6. As referenced in Line 271, the value "n=269/300" corresponds to a calculated result of 89.7%. Please correct this accordingly.
7. In Table 1, the notation "*Scores < 1SD" appears, but the “*” is missing from the table. Please provide an explanation. Additionally, the position of "Spearman’s correlations (r)" is incorrect. Please adjust it accordingly.
8. It is more appropriate to revise Lines 305 and 306 to "with predominantly low to moderate relationships". Additionally, there is an error in the reference to Table 2 mentioned in Line 306 (before revision).
9. Could you please specify the details of the adjustments made to the model in the regression analysis mentioned in Line 333?
