# Supplementary material for: Quality of life in hypertensive patients using the WHOQOL-BREF instrument in the post-pandemic Bangladesh: A cross-sectional study
Source: PLoS One. 2026 Jan 16;21(1):e0340897. doi: 10.1371/journal.pone.0340897 (PMC12810801; doi:10.1371/journal.pone.0340897)
Supplement: Attachment — Submitted filename: Response to Reviewers.docx [file pone.0340897.s009.docx]

**Reviewer Query 1: It is recommended that the title be revised to “Quality of Life in hypertensive patients using the WHOQOL-BREF instrument in the post-pandemic Bangladesh: A cross-sectional study”.**

**Response 1:** Thank you for the constructive suggestion regarding the title. We appreciate the reviewer’s recommendation and agree that the proposed title is clearer and more descriptive. Accordingly, we have revised the manuscript title to:

“Quality of Life in hypertensive patients using the WHOQOL-BREF instrument in post-pandemic Bangladesh: A cross-sectional study.”

This updated title more accurately reflects the study design, target population, and context.

**Reviewer Query 2: References 1 and 2 are listed in reverse order, as are References 24 and 25. Please revise these.**

**Response 2:** We revised the reference orders accordingly in the manuscript.

**Reviewer Query 3: Line 212 states "Those with frequent no daytime napping were considered to have a poor sleep pattern." Are there any relevant references to support this argument?**

**Response 3:** Thank you for pointing this out. The statement in Line 212 contained an unintentional typing error. Our intended definition was that frequent daytime napping, not the absence of napping, is considered an indicator of poor sleep pattern. This definition aligns with existing literature showing that individuals who nap frequently often do so due to inadequate or disturbed nighttime sleep, leading to overall poor sleep quality. Accordingly, we have revised the sentence to:

“Respondents reporting less than 6 (below standard) or more than 8 hours of sleep (Oversleeping), or those with self-reported insomnia or frequent no daytime napping, were considered to have a poor sleep pattern.”

A reference supporting this statement is added in the manuscript.

The correction has now been made in the manuscript.

**Reviewer Query 4: As mentioned in Line 237, the "thirteen-item index" lacks one item in its parenthetical explanation. Please supplement it.**

**Response 4:** 13-item index is enclosed as supplementary file.

**Reviewer Query 5: Table 1 and Table 2 are referenced in reverse order: Table 2 is mentioned in Line 279 while Table 1 is referenced in Line 297. Please correct these.**

**Response 5:** Thank you for your kind feedback. Actually, we rechecked the manuscript and confirmed that the citation of table 1 and table 2 were placed intentionally based on the flow and the contextual chronology in the text. Although the tables are mentioned in the reverse numerical order, each citation correspond to the content being described at that specific point in the paragraph.

**Reviewer Query 6: As referenced in Line 271, the value "n=269/300" corresponds to a calculated result of 89.7%. Please correct this accordingly.**

**Response 6:** Thank you for your careful and critical observation. We corrected that accordingly.

**Reviewer Query 7: In Table 1, the notation "*Scores < 1SD" appears, but the “*” is missing from the table. Please provide an explanation. Additionally, the position of "Spearman’s correlations (r)" is incorrect. Please adjust it accordingly**

**Response 7:** Thank you for your careful review. In Table 1, the superscript notation “a” was intended to indicate that “poor scores” were defined as values <1 SD below the mean; however, due to an oversight, the footnote incorrectly displayed “*Scores <1 SD” instead of “^a^Scores <1 SD”. We have corrected this by replacing the asterisk with the appropriate superscript “^a^” in the footnote so it now matches the table notation. Additionally, we have repositioned “Spearman’s correlations (r)” so that it appears directly above the correlation matrix for proper alignment. These corrections have now been incorporated into the revised table.

**Reviewer Query 8: It is more appropriate to revise Lines 305 and 306 to "with predominantly low to moderate relationships". Additionally, there is an error in the reference to Table 2 mentioned in Line 306 (before revision).**

**Response 8:** Thank you for your valuable insight. We addressed your concern and updated the manuscript accordingly in line 305.

**Reviewer Query 9: Could you please specify the details of the adjustments made to the model in the regression analysis mentioned in Line 333?**

**Response 9:** Thank you. We added a brief elaboration of model adjustment in line 333-334 as-

“Model adjustment was performed using a backward elimination approach to control for potential confounders.”
